# Unsupervised machine learning for clustering forward head posture, protraction and retraction movement patterns based on craniocervical angle data in individuals with nonspecific neck pain

**DOI:** 10.1186/s12891-024-07485-z

**Published:** 2024-05-13

**Authors:** Ui-jae Hwang, Oh-yun Kwon, Jun-hee Kim

**Affiliations:** 1 Department of Physical Therapy, College of Health Science, Laboratory of KEMA AI Research (KAIR), Yonsei University, 234 Maeji-ri, Heungeop-Myeon, Wonju, Kangwon-Do 220-710 Republic of Korea; 2 Department of Physical Therapy, College of Health Science, Laboratory of Kinetic Ergocise Based on Movement Analysis, Yonsei University, Wonju, 26426 Republic of Korea

**Keywords:** Craniocervical alignment, Forward head posture, Neck pain, Unsupervised machine learning, K-means algorithm

## Abstract

**Objectives:**

The traditional understanding of craniocervical alignment emphasizes specific anatomical landmarks. However, recent research has challenged the reliance on forward head posture as the primary diagnostic criterion for neck pain. An advanced relationship exists between neck pain and craniocervical alignment, which requires a deeper exploration of diverse postures and movement patterns using advanced techniques, such as clustering analysis. We aimed to explore the complex relationship between craniocervical alignment, and neck pain and to categorize alignment patterns in individuals with nonspecific neck pain using the K-means algorithm.

**Methods:**

This study included 229 office workers with nonspecific neck pain who applied unsupervised machine learning techniques. The craniocervical angles (CCA) during rest, protraction, and retraction were measured using two-dimensional video analysis, and neck pain severity was assessed using the Northwick Park Neck Pain Questionnaire (NPQ). CCA during sitting upright in a comfortable position was assessed to evaluate the resting CCA. The average of midpoints between repeated protraction and retraction measures was considered as the midpoint CCA. The K-means algorithm helped categorize participants into alignment clusters based on age, sex and CCA data.

**Results:**

We found no significant correlation between NPQ scores and CCA data, challenging the traditional understanding of neck pain and alignment. We observed a significant difference in age (F = 140.14, *p* < 0.001), NPQ total score (F = 115.83, *p* < 0.001), resting CCA (F = 79.22, *p* < 0.001), CCA during protraction (F = 33.98, *p* < 0.001), CCA during retraction (F = 40.40, *p* < 0.001), and midpoint CCA (F = 66.92, *p* < 0.001) among the three clusters and healthy controls. Cluster 1 was characterized by the lowest resting and midpoint CCA, and CCA during pro- and -retraction, indicating a significant forward head posture and a pattern of retraction restriction. Cluster 2, the oldest group, showed CCA measurements similar to healthy controls, yet reported the highest NPQ scores. Cluster 3 exhibited the highest CCA during protraction and retraction, suggesting a limitation in protraction movement.

**Discussion:**

Analyzing 229 office workers, three distinct alignment patterns were identified, each with unique postural characteristics; therefore, treatments addressing posture should be individualized and not generalized across the population.

## Background

While ideal craniocervical alignment has long been considered crucial for musculoskeletal health and rehabilitation, recent research has challenged this traditional view, particularly regarding its relationship with neck pain. Traditionally, abnormal craniocervical alignment, often characterized by a forward head posture (FHP) and measured by the craniocervical angle (CCA), is thought to play a significant role in musculoskeletal discomfort [1, 2]. However, a growing body of evidence suggests a more complex and nuanced connection between craniocervical alignment and neck pain, with some studies finding no clear distinction in CCA between individuals with and without neck pain. This has led to skepticism about the utility of FHP as a primary diagnostic criterion for neck pain [3–5], indicating that the significance of posture in the diagnosis and treatment of neck pain may not be as straightforward as previously believed.

The variations in research findings regarding neck pain and craniocervical alignment highlight the intricate nature of their relationship [6, 7]. Recent studies suggest that the link between these factors is not straightforward, with posture playing a critical role. Specifically, protraction and retraction movements have been identified as key factors influencing neck pain, underscoring the need for a more nuanced approach for diagnosis and treatment [6, 8–10]. Given the frequent presence of cervical protraction in everyday activities and its effectiveness in enhancing head alignment relative to the body and reducing neck discomfort, these factors could be utilized as standards for classifying neck pain [8]. This perspective is supported by evidence indicating that posture adjustments, rather than generic movement or motor control strategies, may offer more effective solutions for managing neck pain [9]. The influence of age and body position (sitting vs. standing) on neck pain further complicates this relationship, suggesting that these factors should be considered in clinical assessments and interventions [1, 11]. Additionally, the co-occurrence of neck pain with headaches, whether as a secondary symptom or due to a primary headache condition [12], highlights the multifaceted nature of neck pain and its management [11]. This refined focus on posture and specific movements aligns with the latest research, suggesting a tailored treatment approach that accounts for individual differences in craniocervical alignment and its impact on neck pain.

Despite substantial progress in understanding movement and posture patterns in individuals with low back pain [13–15], similar research on neck pain remains scarce. Several researchers have reported cases where neck pain has been classified based on movement patterns and motor control [8, 9]. Sahrmann (2010) clearly distinguished the characteristic cervical alignment observed in individuals with cervical flexion and extension syndromes [9]. Characteristic alignment issues among individuals with cervical extension syndrome include increased thoracic kyphosis and FHP, while those among individuals with cervical flexion syndrome include decreased thoracic kyphosis and inward cervical curves [9]. Comerford and Mottram (2012) reported that uncontrolled flexion or extension movements can be confirmed in the cervical spine on an individual basis [8]. However, much of the literature has only studied one FHP pattern and not the various postures and movements linked to neck pain. To address these gaps in knowledge and move beyond standardized therapeutic interventions for neck pain, it is essential to identify and classify the diverse patterns of neck posture and movement among individuals with neck pain.

Clustering analysis can helps classify subjects into clusters using an unsupervised machine learning algorithm [16]. This study used unsupervised machine learning (K-means algorithm) to classify craniocervical alignment patterns based on CCA data in individuals with nonspecific neck pain. The objective of this study was to investigate the association between neck pain and CCA data, categorize craniocervical alignment patterns in individuals with NSNP, and compare CCA data between these patterns and healthy controls.

## Methods

### Study design and participants

This multicenter, retrospective, observational study was conducted at healthcare centers in 11 public service offices. CCA data of office workers (OWs) obtained from musculoskeletal screening tests for preventing industrial accidents were used to examine the risk factors for musculoskeletal disorders in 11 public service offices from April 2022 to February 2023. The OW’s data generated from musculoskeletal screening tests for preventing industrial accidents were used by visiting a musculoskeletal health care program in 11 public service offices. The requirement for informed consent was waived by the Institutional Review Board of Yonsei University Mirae Campus before analysis, as the study used data already acquired by musculoskeletal screening tests to prevent industrial accidents. Personal information was not obtained to protect the anonymity of the participants. A total of 57 OWs without NSNP and 252 OWs with NSNP were screened for eligibility. OWs who had been using computers in the office for more than two years were screened. Individuals with NSNP were included if they (1) reported neck pain intensity over the last month as greater than 3 of 10 on a Numerical Rating Scale (NRS) [17] and (2) had a history of neck pain for more than one month. Individuals without NSNP were eligible if they had no history and experience of neck pain in the last three months. The exclusion criteria for OWs with and without NSNP were diagnosis of tension-type headache, hypertension, rheumatologic conditions, or a history of spinal surgery.

### Craniocervical angle measurements using two-dimensional video analysis

A smartphone equipped with video recording capabilities (4 K resolution, 3840 × 2160 pixels at 60 frames per second) was secured on a tripod positioned 100 cm from the side of the chair, with its height adjusted to align with the level of the participant’s tragus of the ear. Two markers with diameters of 20 mm were attached to the tragus of the ear and spinous process of C7. CCAs during periods of rest, protraction, and retraction were determined by monitoring markers positioned at the lateral canthus of the eye, as well as two additional markers located at the tragus of the ear and spinous process of C7. Python (version 3.6.15; Python Software Foundation) was used to track the markers during protraction and retraction, and OpenCV was used as the main computer vision library. The tracking algorithm uses the Channel and Spatial Reliability Tracking tracker, which reportedly has the most reliable and robust tracking capabilities [18]. The reliability and validity of CCA measurements using two-dimensional video analysis have been a subject of research to ensure the accuracy and consistency of assessments in clinical and research settings [19]. Previous study for reliability and validity of CCA measurements using two-dimensional video analysis suggested that intraclass correlation coefficients (ICCs) for intrarater and interrater reliability ranging from 0.98 to 1.00, indicating excellent reliability. Standard errors of measurement and minimal detectable change values ranged from 0.4° to 0.8° and 0.8° to 2.3°, respectively, suggesting high precision in the measurement of CCA [19].

**Fig. 1:**
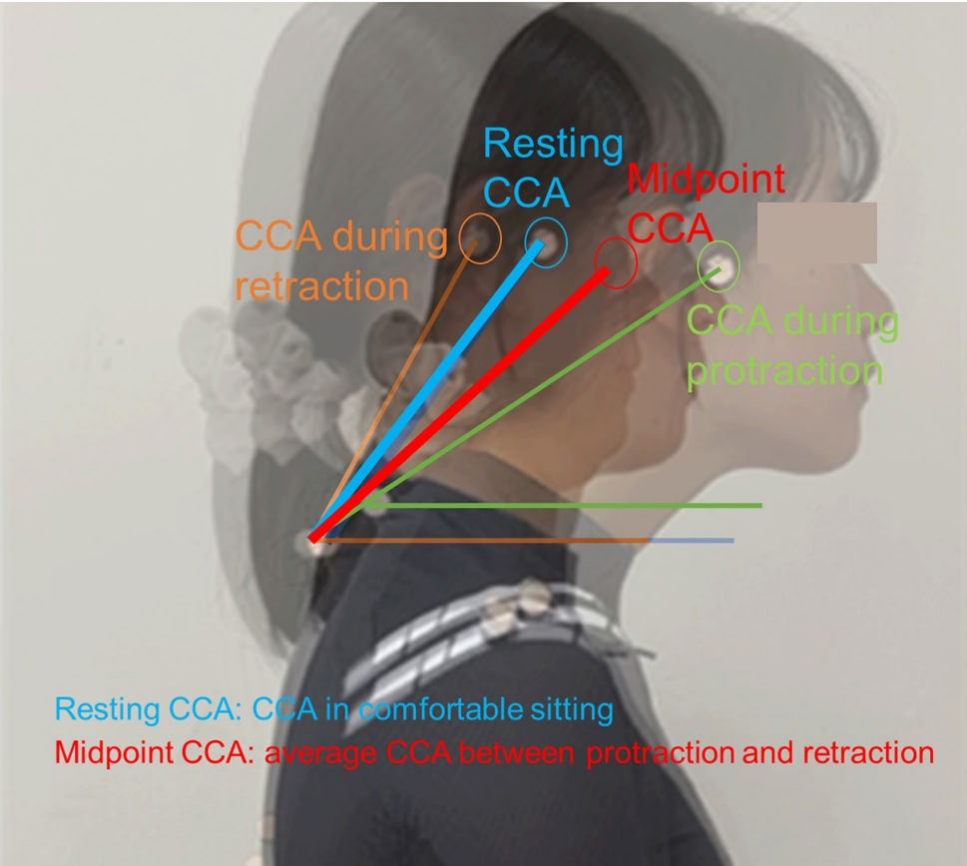
Measurement of resting CCA, CCA during protraction and retraction, and midpoint CCA

CCAs during rest, protraction, and retraction were measured in the sitting position to analyze the resting CCA, midpoint CCA, and difference in CCA between the midpoint and resting CCA (dCCAMR). The midpoint CCA was in the middle position between protraction and retraction for each participant. (Fig. 1). Protraction and retraction were performed consecutively, and each procedure was repeated three times to measure the midpoint CCA. In resting CCA measurements, a decrease in CCA indicates greater FHP. During protraction and retraction, a smaller CCA indicates increased protraction, whereasa larger angle indicates increased retraction.

CCA was measured between the horizontal line passing through the marker on the spinous process of C7 and the line connecting the two markers on the tragus of the ear and the spinous process of C7 (Fig. 1) [20]. The ends of the protraction and retraction were defined as the parts that moved the most in a positive or negative direction from the typical sitting posture on the horizontal axis.

Participants were seated on an adjustable stool without back support, positioned such that the height aligned with their popliteal crease, ensuring a 90-degree angle at both the hips and knees, with feet in a neutral, plantargrade position. Initially, the subjects were instructed to adopt a comfortable and habitual sitting posture, refrain from adjusting their position on the seat, breathe normally, and look forward. This initial posture was used to measure the resting CCA, and was established as the baseline for subsequent protraction and retraction movements. To familiarize themselves with the procedure, the participants performed each movement three times under guidance. For the protraction phase, they were verbally directed to “extend your head forward as much as you can, then return to the baseline posture.” Similarly, for retraction, the instruction was to “pull your head back as far as possible, then return to the starting position”. Both protraction and retraction movements were executed in sequence, and each action was repeated three times to ensure consistency and measurement accuracy.

### Assessing neck pain-related disability Northwick Park Neck Pain Questionnaire

The Northwick Park Neck Pain Questionnaire (NPQ) helps assess neck pain intensity and resulting disability [21]. NPQ includes nine items. Each item consists of five responses. The questions were associated with neck pain intensity, needles/numbness, symptom duration, and various physical activities [21], with scores ranging from 0 to 4 for each question. The maximum possible total score for the test was 36, calculated as a percentage (%). Higher scores indicate greater neck pain intensity and disability.

### Unsupervised machine learning modeling

Machine learning modeling and statistical analysis were performed using Orange data mining software (Orange 3.3.0, Ljubljana, Slovenia) and Python (Version 3.6.15; Python Software Foundation, Wilmington, DE, USA).

#### Pre-processing and missing data handling

Three numeric features are assessed in this study. Out of a total score of 10, scores of 3 or above on the NRS for neck pain and meeting the inclusion criteria were categorized as having NSNP, while scores below 3 and no history and experience of neck pain in the last three months were categorized as not having NSNP. Exploratory data analysis was performed to detect missing data. Imputation for handling missing data was performed by eliminating instances with unknown values. Boxplots were used to assess the distribution of each variable and identify outliers. Outliers were addressed by applying a local outlier factor (contamination = 10%, neighbors = 20, Metric = Euclidean). This process is essential because outliers can significantly affect the accuracy of the learning model.

#### Correlations between NPQ and CCA data

In the entire dataset (*n* = 286), which included both individuals with (*n* = 229) and without (*n* = 57) neck pain, we performed Pearson’s correlation coefficient analyses to explore the relationships between NPQ scores and the CCA data, which encompassed resting CCA, CCA during protraction, CCA during retraction, midpoint CCA, and dCCAMR measurements.

#### Unsupervised machine learning: K-means algorithm

A craniocervical alignment pattern clustering model was developed using the k-means algorithm as an unsupervised machine learning technique. Seven features (age, sex, resting CCA, CCA during protraction, CCA during retraction, midpoint CCA, and dCCAMR) were used for unsupervised machine learning. Because k-means clustering requires pre-determination of the number of clusters before model construction, the number of clusters was set between 2 and 10. The initialization was performed randomly. The number of reruns and the maximum iterations were set to 10 and 300, respectively.

#### Determining optimal number of clusters: silhouette method

In partitioning clustering, determining the appropriate number of clusters, denoted as “h,” can be challenging. Selecting the optimal number of clusters for a given dataset is challenging. One common strategy for finding the optimal number of clusters is calculating the average silhouette score among the criteria used in the optimization process [22]. The silhouette method was used to determine the similarity within the cluster of each data point and the distance between other clusters [22, 23]. The silhouette scores range from 0 to 1, with higher scores indicating better cluster categorization and lower scores indicating poorer cluster categorization [23].

#### Comparisons of NPQ total score and CCA data between clusters and healthy control

Age, NPQ total score, and CCA data (resting CCA, CCA during protraction, CCA during retraction, midpoint CCA, and dCCAMR) between clusters and healthy controls were compared using one-way analysis of variance. A p-value of 0.05 was considered statistically significant. For post-hoc testing, the Bonferroni correction was applied according to the number of comparisons.

## Results

### OWs characteristics

Among the 252 OWs with NSNP, 23 were excluded as outliers to improve cluster classification. The unsupervised machine learning clustering included 229 OWs (14 men and 215 women). The means and standard deviations of all variables are presented in Table 1.

**Table 1:** Mean (standard deviation) of baseline characteristics in OWs with and without NSNP

Variables	Without NSNSP^a^	With NSNP	*p*
Sex (M/F)	14/43	18/233	
Age	36.3 ± 7.1	37.6 ± 6.4	0.231
NRS^b^	0.32 ± 0.70	6.27 ± 1.40	0.000
NPQ^c^ total score (%)	16.33 ± 8.92	46.49 ± 11.59	0.000
CCA^d^ during protraction	32.24 ± 7.21	32.69 ± 7.53	0.680
CCA during retraction	56.94 ± 6.08	56.00 ± 8.05	0.333
Resting CCA	50.08 ± 5.82	49.36 ± 6.36	0.425
Midpoint CCA	44.59 ± 5.71	44.36 ± 6.50	0.779
Difference of CCA between midpoint and rest	-5.49 ± 3.91	-5.02 ± 3.66	0.425

^a^*NSNP* non-specific neck pain, ^b^*NRS* numerical rating scale, ^c^*NPQ* Northwick Park Neck Pain Questionnaire, ^d^*CCA* craniocervical angle

### Correlations between NPQ and CCA data

There were no significant correlations between the NPQ total score and resting CCA (*r* = 0.010, *p* = 0.863, 95% CI=[-0.106, 0.126]), CCA during protraction (*r*=-0.028, *p* = 0.642, 95% CI=[-0.143, 0.089]), CCA during retraction (*r* = 0.061, *p* = 0.303, 95% CI=[-0.055, 0.176]), midpoint CCA (*r*=-0.038, *p* = 0.519, 95% CI=[-0.154, 0.078]), and dCCAMR (*r*=-0.021, *p* = 0.729, 95% CI=[-0.136, 0.096]).

### Number of clusters

**Fig. 2:**
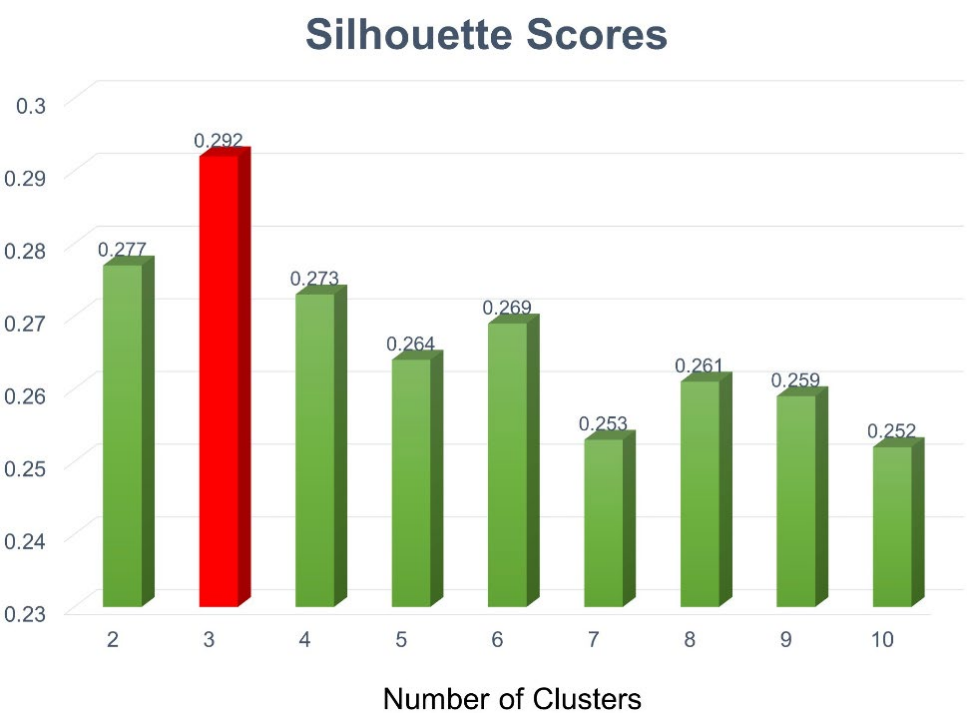
Silhouette scores according to number of clusters

The outcomes of the silhouette method employed with the k-means clustering algorithm are shown in Fig. 2. The silhouette score was the highest when the number of clusters was 3 (silhouette score = 0.292), from 2 to 10. Consequently, the optimal number of clusters for k-means clustering was 3.

### Comparisons of NPQ total score and CCA data between clusters and healthy control

**Fig. 3:**
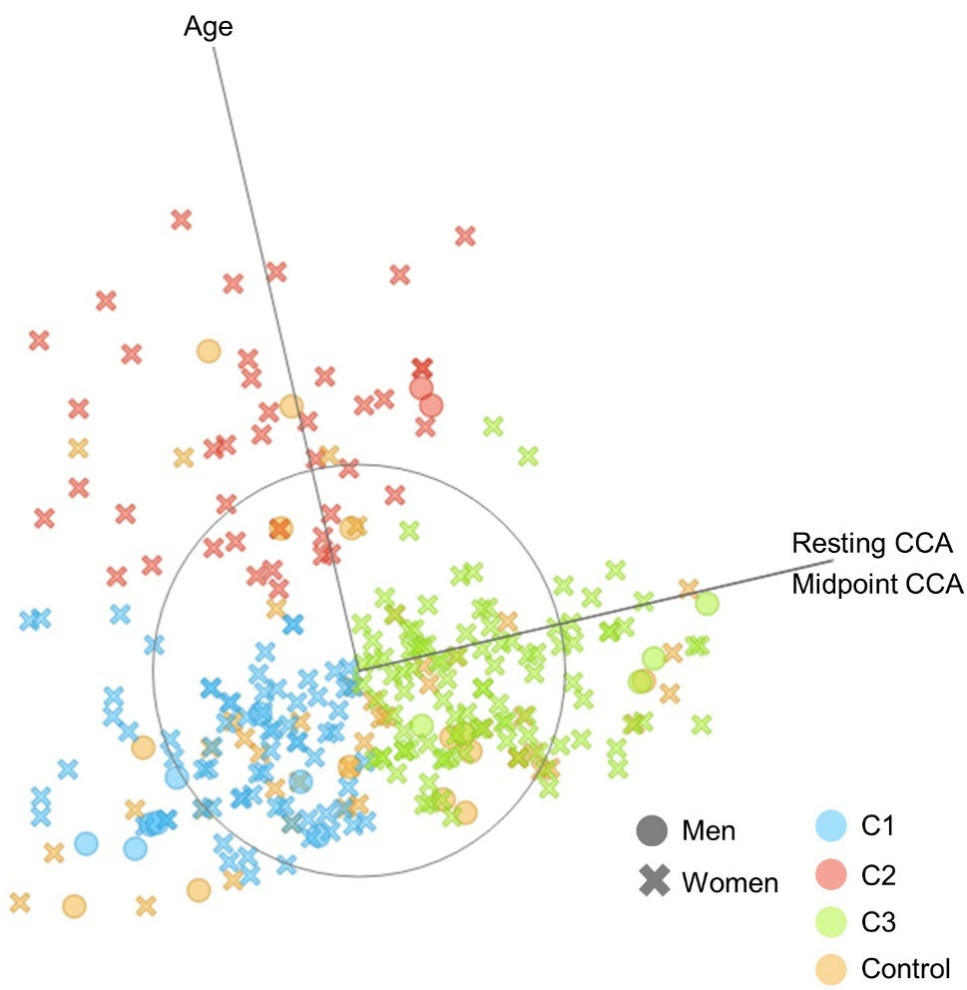
Multi-axis linear projection for OWs with NSNP classified clusters and healthy controls (the greater the dot size, the greater the resting CCA; blue dot = cluster 1; red dot = cluster 2; green dot = cluster 3; orange dot = healthy controls)

**Fig. 4:**
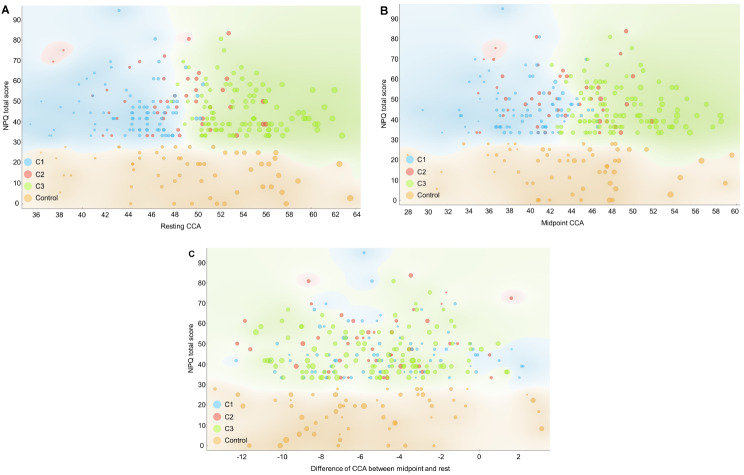
Scatter plot for OWs with NSNP classified clusters and healthy controls. **A**: between resting CCA and NPQ total score, **B**: between midpoint CCA and NPQ total score, **C**: between difference of CCA between midpoint and rest and NPQ total score (the greater the dot size, the greater the resting CCA, blue dot = cluster 1, red dot = cluster 2, green dot = cluster 3, orange dot = healthy controls)

**Fig. 5:**
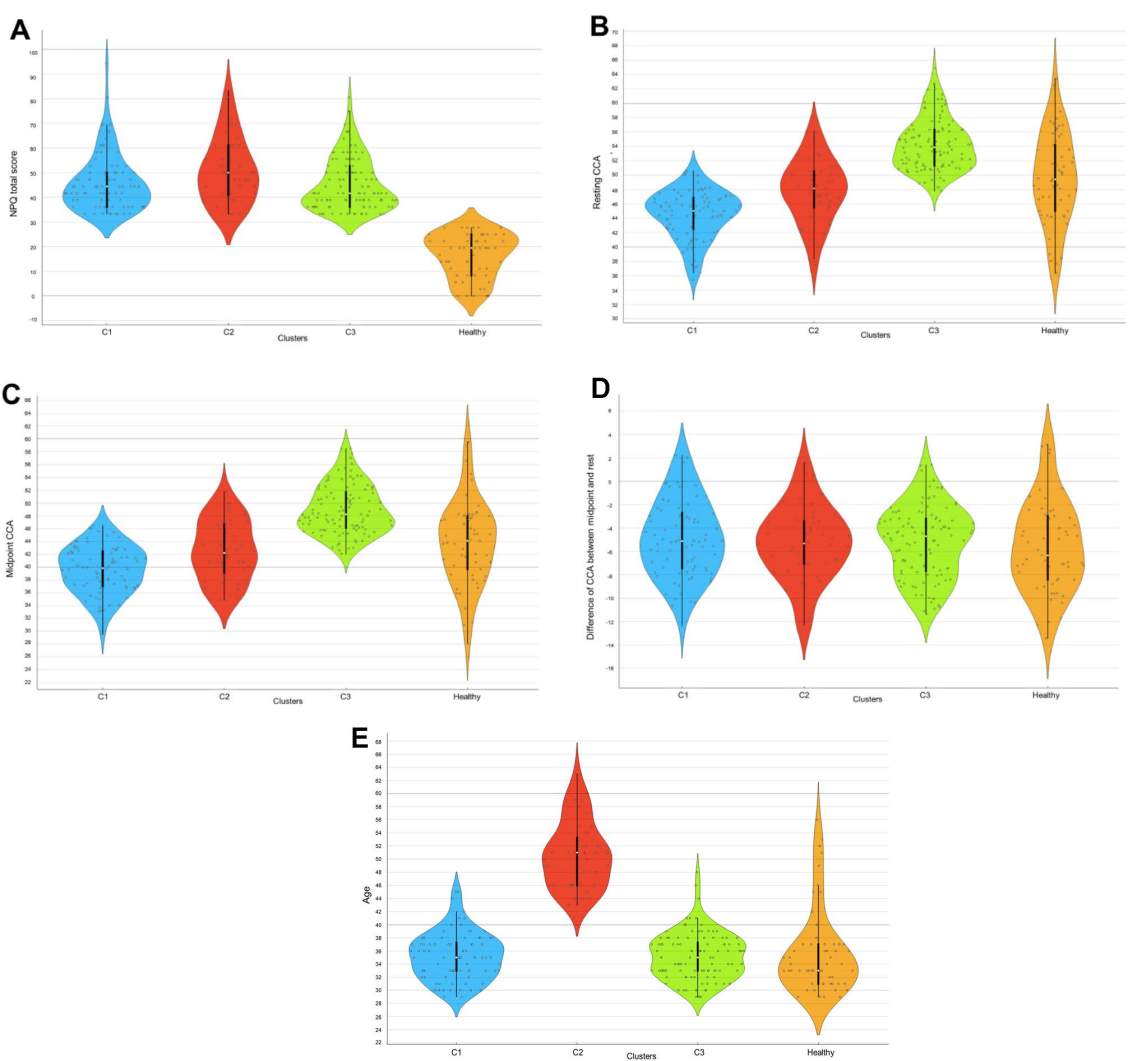
Violin plot for comparisons of CCA data between clusters and healthy control. **A**: NPQ total score, **B**: resting CCA, **C**: midpoint CCA, **D**: difference of CCA between midpoint and rest, **E**: Age

Table 2; Figs. 3 and 4 present the three craniocervical alignment pattern clusters identified using k-means algorithm clustering based on age, sex, resting CCA, CCA during protraction, CCA during retraction, midpoint CCA, and dCCAMR. Figure 3 was expressed using multilinear projection as a type of data visualization used to represent high-dimensional data in two dimensions. The longer the line, the greater the change in the variables between the compared states. The lines can be interpreted as vectors showing the direction and magnitude of change in the data space, which helps in visualizing complex relationships among multiple variables. Figure 4 shows the scatter plot for OWs with NSNP classified clusters and healthy controls between resting CCA, midpoint CCA, dCCAMR and NPQ total score. The distribution of data across clusters can be observed through the violin plot presented in Fig. 5. Among 229 OWs with NSNP, 80, 44, and 104 OWs were classified as clusters 1, 2, and 3, respectively. Significant differences in age (F = 140.14, *p* < 0.001), NPQ total score (F = 115.83, *p* < 0.001), resting CCA (F = 79.22, *p* < 0.001), CCA during protraction (F = 33.98, *p* < 0.001), CCA during retraction (F = 40.40, *p* < 0.001), and midpoint CCA (F = 66.92, *p* < 0.001) were observed among the three clusters and healthy controls. However, there was no significant difference in dCCAMR (F = 0.542, *p* = 0.654).

**Table 2:** Comparisons of CCA data between clusters and healthy controls

Features	Cluster 1 (*N* = 81)	Cluster 2 (*N* = 44)	Cluster3 (*N* = 104)	Healthy controls (*N* = 57)	*p*
Sex	M = 8 / F = 73	M = 2 / F = 42	M = 8 / F = 96	M = 14 / F = 43	-
Age	35.21 ± 3.62	50.64 ± 5.01^*^	35.23 ± 3.56	35.74 ± 6.41	0.000
NPQ^a^ total score (%)	45.80 ± 11.56^*^	50.82 ± 13.25^*^	45.35 ± 10.54^*^	16.32 ± 8.92	0.000
Resting CCA^b^	44.47 ± 3.35^*^	47.84 ± 4.35	54.21 ± 3.52^*^	49.44 ± 6.27	0.000
Midpoint CCA	39.59 ± 3.71^*^	42.54 ± 4.64	48.99 ± 3.78^*^	43.83 ± 6.43	0.000
CCA during protraction	27.81 ± 5.66^*^	31.69 ± 5.40	37.03 ± 5.81^*^	31.33 ± 8.04	0.000
CCA during retraction	51.37 ± 6.24^*^	53.39 ± 6.17	60.94 ± 5.70^*^	56.33 ± 6.48	0.000
Difference of CCA between midpoint and rest	-4.89 ± 3.30	-5.30 ± 3.12	-5.22 ± 3.06	-5.61 ± 3.85	0.654

^a^*NPQ* Northwick Park Neck Pain Questionnaire, ^b^*CCA* craniocervical angle

*p < 0.001 (comparison between cluster vs. healthy control in post-hoc analysis)

For post hoc analysis of age, there was a significant difference in age between groups Cluster 1 and Cluster 2 (*p* < 0.001), with a mean difference of 15.42, suggesting that group Cluster 2 was older than group Cluster 1 (*p* < 0.001). Similarly, a significant difference was observed between Cluster 2 and healthy controls (*p* < 0.001), with Cluster 2 being older. However, no significant age differences were found between groups Cluster 1 and Cluster 3, or Cluster 1 and healthy controls, as well as between Cluster 3 and healthy controls, indicating more homogeneity in age within these groups. For post hoc analysis of NPQ total score, significant differences were observed in NPQT scores between groups Cluster 1 and healthy controls (*p* < 0.001), Cluster 2 and Cluster 3 (*p* < 0.001), Cluster 2 and healthy controls (*p* < 0.001), and Cluster 3 and healthy controls (*p* < 0.001), suggesting variations in neck pain experiences across different groups. No significant difference was observed between groups Cluster 1 and Cluster 2, or Cluster 1 and Cluster 3. For both resting CCA and midpoint CCA variables, all group comparisons except Cluster 2 and healthy controls for resting CCA and midpoint CCA showed significant differences (*p* < 0.001). For CCA during retraction, significant differences were notably present between groups Cluster 1 and Cluster 3 (*p* < 0.001), Cluster 1 and healthy controls (*p* < 0.001), Cluster 2 and Cluster 3 (*p* < 0.001), and Cluster 3 and healthy controls (*p* < 0.001). Similarly, CCA during protraction showed significant variability, with differences observed between groups Cluster 1 and Cluster 2 (*p* < 0.001), Cluster 1 and Cluster 3 (*p* < 0.001), Cluster 1 and healthy controls (*p* < 0.001), Cluster 2 and Cluster 3 (*p* < 0.001), and Cluster 3 and healthy controls (*p* < 0.001).

## Discussion

Physical therapists and healthcare practitioners frequently conduct craniocervical alignment analyses as part of their screening tests, which can provide valuable insights into the presence of NSNP [5, 24]. Recent studies, aligning with our results, have shown weak, non-significant correlations between CCA and clinical measures of pain and disability, challenging the historical emphasis on FHP as a primary cause of neck pain [3–5]. In line with these recent studies, our study could not confirm a significant correlation between neck pain and craniocervical alignment and movement, or significant differences in craniocervical alignment and movement between individuals with and without NSNP. These results suggest a non-linear relationship between neck pain and craniocervical alignment, indicating that clinical assessment strategies and the development of targeted interventions may be crucial for NSNP. The identification of three distinct craniocervical alignment patterns among individuals with NSNP challenges the conventional understanding that greater FHP is primarily associated with neck pain severity. Our findings could suggests the variability in neck pain etiology and the limitations of a one-size-fits-all approach for diagnosis and treatment. We identified three distinct craniocervical alignment patterns among individuals with NSNP, each with unique characteristics and implications. The present study could indicat the need for a more personalized approach to treatment for NSNP, taking into account the specific alignment patterns and movement restrictions of each individual.

Individuals classified as Cluster 1 accounted for 35.4% of 229 OWs with the NSNP. The resting and midpoint CCAs in Cluster 1 were significantly lower than those in the healthy controls. Cluster 1 had the lowest resting CCA, i.e., the FHP increased the most among these cases. Cluster 1 exhibited the least CCA during retraction within the three clusters and healthy controls. Decreased cervical retraction movement could be attributed to tightness of the sternocleidomastoid and posterior neck muscles, insufficient mobility of the cervical spine, and weakness of the deep neck flexors as cervical stabilizers [25, 26]. FHP affects the cervical spine by imposing increased mechanical load, altering cervical mobility, and leading to overuse of muscles such as the scapular elevators, sternocleidomastoid, and upper trapezius [27, 28]. Thus, OWs with NSNP, including those in Cluster 1, could be classified as exhibiting a pattern indicative of restriction of retraction movement and increasing FHP.

Of the 229 OWs with NSNP, individuals classified as belonging to Cluster 2 comprised 19.2% of the total. Cluster 2 individuals were characterized by being the oldest among the clusters, with an age significantly higher than that of Cluster 1, Cluster 3, and healthy controls. Despite this age difference, Cluster 2’s CCA data (including resting CCA, CCA during protraction, and CCA during retraction) showed no significant differences from healthy controls. This observation suggests that cervical posture and movement may not be directly related to the NSNP in this particular cluster. This aligns with some studies suggesting a complex and not always straightforward relationship between cervical posture, movement, and NSNP. These findings suggest that the etiology of NSNP, particularly in Cluster 2, might not be primarily driven by observable postural deviations or specific movement patterns but rather by a complex interaction of factors, including age-related changes, muscle function, and perhaps other not yet fully understood biomechanical or physiological factors [5, 29, 30]. The lack of significant difference of CCA data between Cluster 2 and healthy controls underscores the imperative for a comprehensive diagnostic and therapeutic strategy for NSNP. This strategy should extend beyond mere assessment of physical posture and movement patterns to encompass a variety of potential contributing factors. Such factors include, but are not limited to, psychological stressors, muscular endurance capabilities, and the influence of aging on musculoskeletal functionality [31, 32]. This multifaceted approach is essential for a more nuanced understanding and effective management of NSNP. Furthermore, Cluster 2 exhibited the highest NPQ total scores statistically, despite not showing significant differences in posture or movement compared to healthy controls. Given the advanced age of this cluster, age-related physiological changes, decreased muscular endurance, or psychological factors such as the perception of pain and disability may play a more significant role in the manifestation of NSNP in these individuals [31]. Alternatively, it is conceivable that individuals experiencing pain due to incorrect posture or movements might adaptively modify their posture and movement patterns to avoid pain, thereby aligning closer to those observed in healthy controls [33]. The concept of pain avoidance through adaptive postural adjustment is supported by the pain adaptation model, which posits that pain can lead to changes in motor control strategies as a protective mechanism to minimize discomfort and prevent further injury [33, 34]. This theory is further corroborated by studies suggesting that individuals with chronic pain may develop compensatory movement patterns to mitigate pain, potentially masking underlying postural deviations or movement dysfunctions when compared to asymptomatic individuals [35, 36]. These findings could suggest the importance of a nuanced approach to evaluating and treating NSNP, considering not only the mechanical aspects of posture and movement but also the individual’s adaptive responses to pain.

Among 229 OWs with NSNP, individuals classified as Cluster 3 accounted for 45.4%. The resting and midpoint CCAs in Cluster 3 were significantly greater than those in the healthy controls. Cluster 3 showed the greatest CCA during protraction (i.e., protraction decreased) among the three clusters. A previous study reported that retraction showed a significantly greater range in the neck pain group than in the healthy group [37]. This may be because excessive extension movements occur in a specific segment of the lower cervical spine, resulting in retraction rather than protraction. Furthermore, limitations in protraction movements might have arisen from concerns about the potential pain associated with protraction. Retractions can potentially facilitate cervical root decompression and reduce radicular pain, which could influence the preference for retraction over protraction in certain cases [38]. Thus, OWs with NSNP, including Cluster 3, could be classified as a pattern for restricting protraction movement.

The clinical implications of our findings are significant, particularly in the context of personalized assessments and treatment strategies for NSNP. The identification of specific craniocervical alignment patterns among individuals with NSNP suggests that clinicians should consider these patterns when developing treatment plans. For instance, individuals categorized within Cluster 1, which is characterized by a pattern of restricted retraction movement, may benefit more from targeted exercises designed to improve lower cervical extension and upper cervical flexion mobility. This recommendation is supported by studies emphasizing the effectiveness of customized physical therapy interventions based on individual posture analysis [11, 37]. For Cluster 3, which exhibits a pattern of restricted protraction movement, interventions could prioritize exercises that enhance cervical protraction mobility and manage excessive retraction, aligning with evidence suggesting the benefits of specific movement strategies in treating neck pain [8, 9]. Thus, our study underscores the importance of integrating detailed posture and movement pattern analysis into clinical practice, advocating for a model of care that is responsive to the individual characteristics of each patient with NSNP.

Although our study yielded valuable insights, it was not without its limitations. First, the study population was restricted to OWs, which hinders the generalizability of the findings to a broader population. Further research with larger and more diverse sample sizes is needed to extend the applicability of these results. Second, the study was cross-sectional. As a result, we could not establish causal relationships; we could only observe associations and correlations among the variables at a specific time. Longitudinal studies are required to explore the cause-and-effect relationships more definitively. The potential influence of these alignment and movement patterns on treatment outcomes must be explored. Furthermore, longitudinal studies would help to establish causal relationships between these patterns and the development or persistence of neck pain. Future studies should investigate whether targeted interventions tailored to specific alignment patterns can improve the clinical outcomes. Third, the k-means algorithm we chose might not necessarily be the best-performing model given the diverse machine learning algorithms available. Fourth, our analysis of posture was confined to a single plane, overlooking the three-dimensional nature of posture. This limitation restricts our understanding of the complex spatial orientation of the cervical spine and its contribution to neck pain. Future research should incorporate multi-planar analyses to capture the full scope of postural dynamics. Fifth, the study did not differentiate between mobility and function of the upper versus the mid/lower cervical spine, which are known to vary among individuals. Our measurement of only one angle in the craniocervical alignment failed to identify where movement predominantly occurred within the cervical spine of an individual participant. For instance, it remains unclear whether the upper cervical spine extendeds more than the lower cervical spine during protraction. This oversight suggests that there could be significant variations in how participants positioned each region of their cervical spine across the measured postures.

## Conclusion

This study underscores the complexity of the association between craniocervical alignment and neck pain. We identified distinct craniocervical alignment patterns in individuals with neck pain by applying the k-means algorithm as an unsupervised machine learning technique. These findings open new avenues for personalized assessment and treatment, potentially revolutionizing the management of NSNP. Further research is needed to validate and expand these findings, ultimately leading to more effective interventions in individuals with neck pain.

## Data Availability

The datasets analyzed during the current study are available from the corresponding author on reasonable request.

## Abbreviations

CCA: Craniocervical angle
dCCAMR: Difference in CCA between Midpoint and Resting
FHP: Forward head posture
NRS: Numerical Rating Scale
NPQ: Northwick Park Neck Pain Questionnaire
NSNP: Nonspecific neck pain
OW: Office workers
